# Investigation of azole resistance in Aspergillus species isolated from clinical specimens by azole agar screening method

**DOI:** 10.1099/jmm.0.002112

**Published:** 2026-01-13

**Authors:** Zeynep Yazgan, Gökhan Aygün, Selçuk Ahmet Algıngil, Reyhan Caliskan

**Affiliations:** 1 Department of Medical Microbiology, Cerrahpasa School of Medicine, Istanbul University-Cerrahpasa, Istanbul, Turkey; 2 Department of Infectious Diseases and Clinical Microbiology, Cerrahpasa School of Medicine, Istanbul University-Cerrahpasa, Istanbul, Turkey; 3 Istanbul Aydin University Department of Food Safety, Fullgen Biotechnology Industry Trade Limited, Istanbul, Turkey; 4 Department of Medical Microbiology, Samsun University Faculty of Medicine, Basic Medical Sciences, Samsun, Turkey

**Keywords:** agar plate method, *Aspergillus*, azole resistance, cypA-L98H, cypA-M220, *cyp51A*

## Abstract

**Introduction.** Aspergillosis represents a significant global health threat, with increasing concerns about azole resistance.

**Hypothesis/Gap Statement.** There is limited evidence on the prevalence and distribution of azole resistance among clinical *Aspergillus* isolates.

**Aim.** This study investigated the prevalence of azole resistance in clinical *Aspergillus* isolates and evaluated different susceptibility testing methods.

**Methodology.** A total of 125 *Aspergillus* spp. isolates were collected from clinical samples (abscess, corneal abscess, biopsy, tissue and respiratory samples). Species identification was performed using conventional morphological methods and Matrix assisted laser desorption Ionization time of flight massspectrometry (MALDI-TOF) MS. Azole susceptibility testing was conducted using the gradient test (E-test), the agar plate screening method and broth microdilution for voriconazole (VOR), itraconazole (ITR) and posaconazole (POS). Molecular analysis was performed to detect *cyp51A* gene mutations associated with resistance.

**Results.** Among 125 isolates, species distribution was 44% *Aspergillus fumigatus*, 33.6% *Aspergillus flavus*, 5% *Aspergillus terreus*, 3% *Aspergillus niger* and 14% *Aspergillus* spp. Using gradient testing, *A. fumigatus* showed 1.8% resistance to VOR, 5.45% to ITR and 1.8% to POS, with one isolate resistant to all azoles. *A. terreus* showed 16.7% resistance to VOR, *A. niger* 25% resistance to ITR and *Aspergillus* spp. showed various resistance patterns. The agar plate method detected resistance with 100% susceptibility/specificity for *non-fumigatus* species but 33.3% susceptibility for *A. fumigatus* ITR resistance. CypA-L98H mutations were detected in six isolates and CypA-M220 mutations in seven isolates.

**Conclusion.** This study confirms the presence of azole resistance in clinical *Aspergillus* isolates with species-specific variations. The agar plate screening method shows promise for *non-fumigatus* species but requires optimization for *A. fumigatus*.

## Summary

This study reported azole resistance in 125 clinical *Aspergillus* isolates, finding 5.6% overall resistance with species-specific variations. Agar plate screening showed 100% susceptibility/specificity for *non-fumigatus* species but only 33.3% susceptibility for *Aspergillus fumigatus* itraconazole resistance.

## Introduction

Aspergillosis, a fungal infection caused by *Aspergillus* species, has been increasingly observed among immunocompromised populations and is associated with elevated mortality rates. The growing prevalence of such fungal pathogens has become a significant global public health concern, prompting the World Health Organization to designate them as a priority issue and classify *Aspergillus fumigatus* as a critical threat on their priority pathogen list [1]. As the most common mould infection in immunocompromised patients, aspergillosis presents additional challenges due to the emergence of drug resistance in *Aspergillus* spp., which has become a major concern in clinical practice.

Invasive aspergillosis, caused by *A. fumigatus* that has developed resistance to azoles, is characterized by high mortality rates [2]. Azole antifungals are the first-line treatment for these life-threatening infections. Recently, azole resistance in *A. fumigatus* has increasingly been reported as a cause of treatment failure [3]. Invasive aspergillosis has become a global health threat, exacerbated by the rapid increase in antifungal resistance and limited access to high-quality diagnosis and treatment in many settings. Careful use of azole antifungals for prophylactic and therapeutic purposes, as well as investigation of resistance through *in vitro* susceptibility testing where appropriate, is essential [4]. Compared to *A. fumigatus*, there is limited information on the frequency and mechanisms of azole resistance in *non-fumigatus Aspergillus* isolates [5,6]. Additionally, the effectiveness of agar plate screening for detecting azole resistance in these species remains unclear.

Therefore, there is a need to develop simple and practical screening methods for identifying resistance in both azole-resistant *A. fumigatus* and *non-fumigatus Aspergillus* species in clinical mycology laboratories. This study investigated the applicability of agar plate screening as a reliable method for detecting antifungal resistance in *Aspergillus* spp.

## Methods

This study included various isolates identified as *Aspergillus* spp. obtained from different clinical samples (abscess, corneal abscess, biopsy, tissue and respiratory samples) at the Medical Microbiology Mycology Laboratory of Cerrahpaşa Faculty of Medicine Hospital between 2015 and 2021. In instances where multiple isolates were procured from successive samples derived from the same patient, only the initial isolate was incorporated into the investigation. Patient information was acquired from the hospital information system (ISHOP). The research protocol was approved by the Clinical Research Ethics Committee of Istanbul Aydın University (Decision No: 2022/66).

### Identification of *Aspergillus* spp. isolates by conventional methods

*Aspergillus* spp. isolates were grown on Sabouraud dextrose agar (SDA; HiMedia) and potato dextrose agar (PDA; Difco), incubated at 25 and 35 °C. Growth was monitored after 24, 48 and 72 h. Isolates were identified based on both macroscopic/microscopic morphological attributes, particularly their ability to grow at different temperatures, including 45 °C (thermotolerance test). For microscopic identification, a portion of the colony grown on PDA, including its peripheral zone, was examined by the cellophane tape method and lactophenol cotton blue staining. *Aspergillus niger* showed growth within 24 h, whereas other *Aspergillus* species were examined for growth after at least 48 h [7,8]. Isolates that were not discerned using conventional methodologies were identified by MALDI-TOF MS (MALDI/Biotyper, Bruker Daltonik GmbH) in accordance with the prescribed protocol. Isolates that could not be identified by either method were designated as *Aspergillus* spp.

### Antifungal susceptibility testing for azole resistance

Azole susceptibility testing of *Aspergillus* spp. voriconazole (VOR), itraconazole (ITR) and posaconazole (POS) was performed using the gradient test (GT; E-test), the agar screening plate method and broth microdilution (BMD). *Aspergillus* spp. isolates were incubated with SDA at 35 °C for 2–7 days to ensure sufficient sporulation. Isolates resistant to the gradient or agar plate method were retested using the BMD method for confirmation. Antifungal susceptibility tests were repeated if resistance was detected in all methods.

GT (E-test): *Aspergillus* spp. isolates were prepared to match a McFarland turbidity standard (0.5) using RPMI 1640 medium (Sigma Chemical Co., St. Louis, MO, USA) buffered with MOPS (Sigma Chemical Co., St. Louis, MO, USA) [Bacto agar and D glucose (Oxoid)]. E-test strips (VOR, ITR and POS; Biomerieux, France) were then placed on the medium. The plates were then incubated at 35 °C for a period of 24–48 h. Antifungal susceptibility testing (AFST) by GT was evaluated at 48 h for *A. fumigatus*, *Aspergillus flavus* and *A. niger*; at 48 h for *Aspergillus* spp. and *Aspergillus terreus*; and at 72 h for some rare slow-growing *Aspergillus* spp. Test results were interpreted using clinical breakpoint values for antifungal epidemiological cut-off values (ECOFFs) and susceptibility according to EUCAST procedures (EUCAST E.Def7.4, E.Def9.4, E.Def11.0). The MIC50/90 values were determined for each antifungal agent [9].

Agar screening plate method: Following EUCAST recommendations, RPMI 1640 (with 2% glucose) was supplemented with ITR (4 mg l^−1^), VOR (2 mg l^−1^) and POS (0.5 mg l^−1^), with drug-free medium used as a control [9,12]. *Aspergillus* spp. conidial suspensions [0.5 McFarland (25 µl)] were inoculated onto RPMI 1640 (2% glucose) plates containing VOR, ITR, POS and control plates, followed by incubation at 35 °C for 48 h. Test results were scored by determining the presence or absence of growth on plates containing VOR, ITR and POS. Growth in the presence of a drug of interest was interpreted as resistance. The susceptibility/specificity of the agar screening plate method for the detection of azole resistance was calculated using the GT method.

BMD method: AFST was performed using the CLSI M38/A2 reference BMD method. Stock solutions of the antifungals ITR (Sigma Chemical Co., St. Louis, MO, USA), VOR (UK-109, 496; Vfend, Pfizer Pharmaceuticals, New York) and POS (Sigma Chemical Co., St. Louis, MO, USA) were prepared at 1,600 µg ml^−1^ in DMSO (Merck KGaA, Germany). Working concentrations were prepared by serial dilution with RPMI 1640 (l-glutamine, bicarbonate-free, Sigma Chemical Co., St. Louis, MO, USA), resulting in a final antifungal concentration of 0.032–16 µg ml^−1^. In each well of the U-bottom microdilution plates, 100 µl of each antifungal dilution was added, and 100 µl of 0.5 McFarland fungal suspension was added to each well. The plates were incubated at 35 °C for 24/48 h. According to EUCAST procedures, test results were interpreted using clinical breakpoints and antifungal ECOFFs [8]. MIC50/90 values were calculated for each antifungal agent. Known control strains, *Candida krusei* ATCC 6258 and *Candida parapsilosis* ATCC 22019, were used for quality control of AFST.

### Identification of azole-resistant *Aspergillus *spp. isolates by DNA sequencing

To identify the azole-resistant *Aspergillus* isolates, DNA sequencing was conducted using the primers from Sentromer DNA Technologies (Istanbul, Turkey) to amplify specific gene regions.

ITS1-F (5′-TCCGTAGGTGAACCTGCGG-3′) and

ITS4-R (5′-TCCTCCGCTTATTGATATGC-3′) primers.

The ITS1-5.8S-ITS2 region of fungal rRNA was amplified by PCR for Sanger sequencing, and 2X PCRBIO HS Taq Mix (PCR Biosystems Ltd., London, England) was used as a DNA template for each of the universal fungal primers ITS1 and ITS4 (CDC, 2024).

### Detection of mutations in the *Cyp51A* gene

Azole resistance-associated mutations in the *Cyp51A* gene (GenBank accession number AF338659.1) in *Aspergillus* spp. isolates were analysed using quantitative real-time PCR (qPCR) using the commercial MicroLine SYBR GreenMasterMix (2×) kit (Fullgen Biotechnology Industry and Trade Ltd., Turkey). Primer sets were specifically designed to target key mutation sites L98H and M220 in the *Cyp51A* gene, which are clinically important for azole resistance profiling [13]. Melt curve analysis was performed from 72 to 95 °C with increments of 0.3 °C per second to assess amplicon specificity and identify mutation-specific Tm shifts [14].

CypA-L98H-F (5′-AAAAAACCACAGTCTACCTGG-3′) and

CypA-L98H-R (5′-GGAATTGGGACAATCATACAC-3′),

CypA-M220-F (5′-GCCAGGAAGTTCGTTCCAA-3′) and

CypA-M220-R (5′-CTGATTGATGATGTCAACGTA-3′) primers.

### Statistical analysis

Statistical analysis was performed using Fisher’s exact test to compare proportional differences between categorical groups. This method was specifically chosen over the Chi-square test due to the low sample sizes and small expected cell frequencies (e.g. *n*<5) observed in the data, which can invalidate the assumptions of the Chi-square test. Fisher’s exact test was applied to 2×2 contingency tables to: evaluate the performance of the agar plate screening method against the GT for detecting ITR resistance in *A. fumigatus*. Compare the prevalence of ITR resistance between *A. fumigatus* and *A. niger*. Compare the prevalence of VOR resistance between *A. fumigatus* and *A. terreus*. A *P* value of less than 0.05 was considered statistically significant.

## Results

### Characteristics of *Aspergillus* spp. isolates

In this study, 125 *Aspergillus* spp. isolates that met these criteria were included in further analysis (Table 1). Among the patients with these isolates, 56.8% (*n*=71) were male, 43.2% (*n*=54) were female and 21.6% (*n*=27) were children. The age range was 3–82 years, and the average age was 44. Of the 125 isolates, 56.8% (*n*=71) were isolated from the respiratory tract [bronchoalveolar lavage (BAL), endotracheal aspiration (ETA), sputum and nasal sinus contents], 20% (*n*=25) from tissue, 13.6% (*n*=17) from abscess, 5.6% (*n*=7) from corneal abscess and 4% (*n*=5) from other samples (body fluids such as pus, pleura, catheter, etc.). Isolates growing at 45 °C by thermotolerance test were identified as *A. fumigatus sensu stricto*. Using conventional methods or MALDI-TOF MS, 44% *A*. *fumigatus* (*n*=55), 33.6% *A*. *flavus* (*n*=42), 5% *A*. *terreus* (*n*=6), 3% * A*. *niger* (*n*=4) and 14% *Aspergillus* spp. (*n*=18) were identified.

**Table 1.** Distribution of clinical samples in which *Aspergillus* spp. isolates were produced

Category	Subcategory	Count (*n*)	Percentage (%)
**Patient demographics**	Male	71	56.8
	Female	54	43.2
	Children	27	21.6
**Sample source**	Respiratory tract	71	56.8
	Tissue	25	20.0
	Abscess	17	13.6
	Corneal abscess	7	5.6
	Other samples	5	4.0
**Species identification**	*A. fumigatus*	55	44.0
	*A. flavus*	42	33.6
	*Aspergillus* spp.	18	14.0
	*A. terreus*	6	5.0
	*A. niger*	4	3.0

### Azole susceptibility results of *Aspergillus* spp. isolates via AFST

#### Azole susceptibility results of *Aspergillus* spp. isolates using the GT

Using the GT method, resistance was detected in 1 (1.8%) of 55 *A*. *fumigatus* isolates to VOR, in 3 (5.45%) isolates to ITR and in 1 (1.8%) isolate to POS. One isolate (1.8%) was resistant to all the azoles. Among the six *A*. *terreus* isolates, one (16.7%) exhibited a VOR-MIC of 1 µg ml^−1^. Among the four *A*. *niger* isolates, one (25%) had an ITR-MIC of 2 µg ml^−1^. Among 18 *Aspergillus* spp. isolates, 1 (5.6%) had a VOR-MIC of 1.5 µg ml^−1^, 1 (5.6%) had a VOR-MIC >32 µg ml^−1^, 1 (5.6%) had an ITR-MIC of 4 µg ml^−1^ and 2 isolates (11%) had POS-MIC values of 0.5 and 8 µg ml^−1^.

**Fig. 1.**
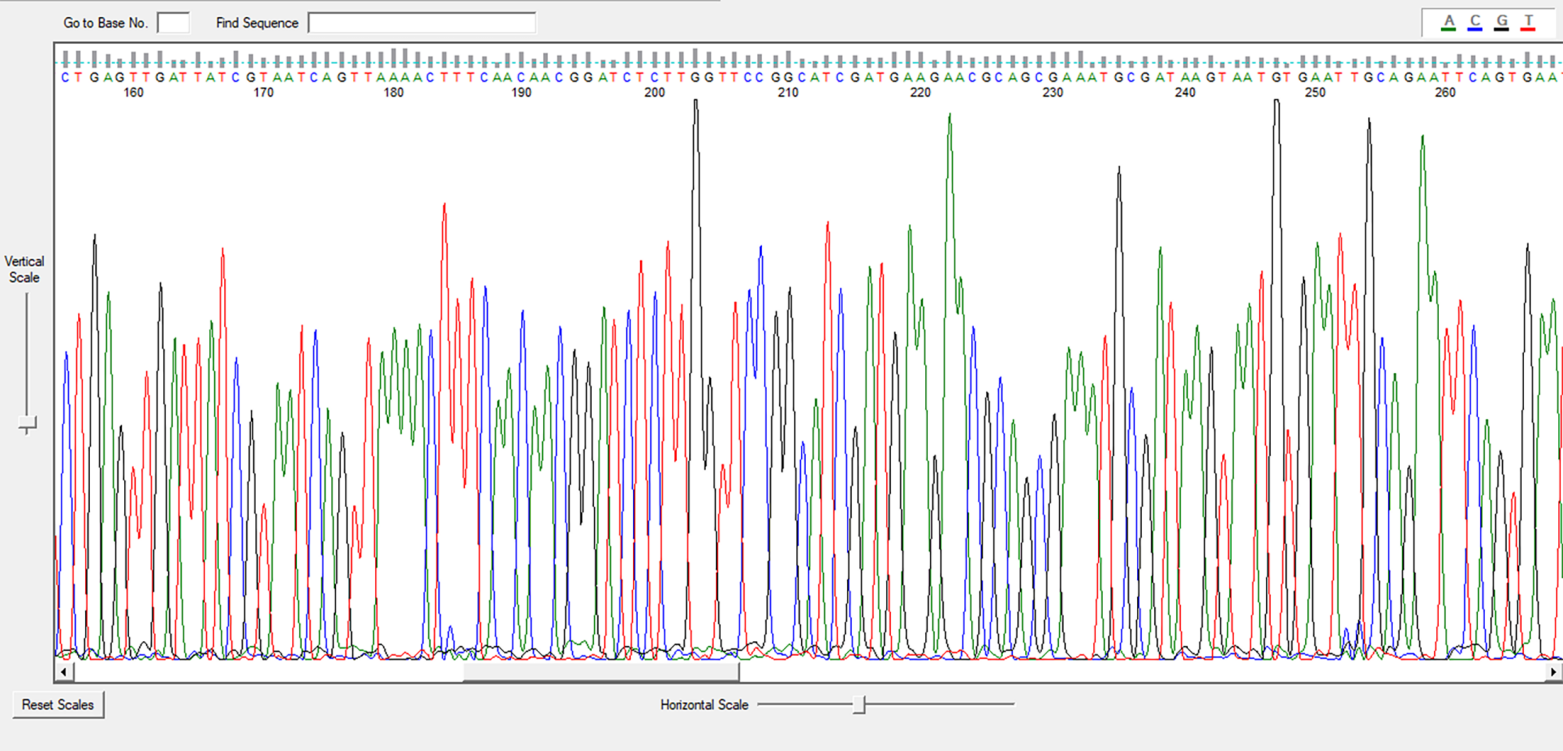
*A. fumigatus* sample diagram.

Gradient testing identified resistance in: the overall azole resistance rate was 5.6%, with specific resistance patterns observed: * A. fumigatus*: 1.8% (*n*=1) resistant to VOR and POS, 5.45% (*n*=3) resistant to ITR. *A. terreus*: 16.7% (*n*=1) resistant to VOR-MIC: 1 µg ml^−1^. *A. niger*: 25% (*n*=1) resistant to ITR-MIC: 2 µg ml^−1^. *Aspergillus* spp.: 5.6% (*n*=1) resistant to ITR-MIC: 4 µg ml^−1^, 11% (*n*=2) resistant to VOR and POS (MICs: 1.5 µg ml^−1^, >32 µg ml^−1^, 0.5 µg ml^−1^ and 8 µg ml^−1^). The GT plates are shown in Fig. 1, and the MIC50/MIC90 values are listed in Table 2. 

**Table 2.** MIC50 and MIC90 values of azole antifungals for *Aspergillus* spp. isolates detected via GT

	Antifungal	MIC range (µg ml^−1^)	MIC50 (µg ml^−1^)	MIC90 (µg ml^−1^)
***A. fumigatus* complex (*n*=55**)	VOR	0.032–>32	**0.19**	**0.38**
	ITR	0.19–4	**0.5**	**1**
	POS	<0.002–0.75	**0.094**	**0.19**
***A. flavus* complex (*n*=42)**	VOR	0.047–0.75	**0.19**	**0.38**
	ITR	0.125–1	**0.38**	**0.75**
	POS	<0.002–0.19	**0.064**	**0.125**
***A. terreus* (*n*=6)**	VOR	0.023–1	**0.064**	**0.125**
	ITR	0.125–1	**0.19**	**0.5**
	POS	0.012–0.19	**0.032**	**0.064**
***A. niger* (*n*=4)**	VOR	0.094–0.5	**0.5**	**0.5**
	ITR	0.5–2	**0.75**	**0.75**
	POS	0.012–0.19	**0.19**	**0.19**
***Aspergillus* spp*.* (*n*=18)**	VOR	0.032–>32	**0.125**	**0.5**
	ITR	0.047–4	**0.5**	**0.75**
	POS	<0.002–8	**0.094**	**0.19**

#### Azole resistance in *Aspergillus* spp. isolates detected by agar plate method

Using the agar plate method, resistance was detected in 1 (1.8%) of 55 *A. fumigatus* isolates to VOR, ITR and POS; in 1 (16.7%) of 6 *A. terreus* isolates to VOR; in 1 (25%) of 4 *A. niger* isolates to ITR; in 2 (11%) of 18 *Aspergillus* spp. isolates to VOR-POS; and in 1 (5.6%) isolate to ITR. All the 42 *A. flavus* isolates were susceptible to VOR, ITR and POS. The susceptibility and specificity for VOR-POS in *A. fumigatus* were 100% and 33.3 and 100% for ITR, respectively. For *A. flavus*, the ITR susceptibility was 100%, whereas for *A. terreus*, the ITR and POS sensitivities were 100%.

Agar plate testing detected resistance in: *A. fumigatus*: 1.8% (*n*=1) resistant to VOR, ITR and POS; *A. terreus*: 16.7% (*n*=1) resistant to VOR; *A. niger*: 25% (*n*=1) resistant to ITR. *Aspergillus* spp.: 11% (*n*=2) resistant to VOR and POS; 5.6% (*n*=1) resistant to ITR. The susceptibility and specificity of the agar plate method varied: for ITR resistance in *A. fumigatus*: susceptibility=33.3%. For ITR, VOR and POS resistance in *non-fumigatus Aspergillus*: susceptibility and specificity=100%. The agar plate method exhibited 100% susceptibility and specificity for detecting azole resistance in *non-fumigatus Aspergillus* species, but its susceptibility for ITR resistance in *A. fumigatus* was only 33.3%.

#### BMD results of *Aspergillus* spp. isolates

By BMD method in seven isolates in which resistance was detected by GT and/or agar plate screening method: *A. fumigatus* (*n*=1) is resistant to ITR, VOR and POS; *A. fumigatus* (*n*=2) is resistant to ITR; *A. terreus* (*n*=1) ITR-MIC: 1 µg ml^−1^ and VOR-MIC: 0.5 µg ml^−1^; *A. niger* (*n*=1) ITR-MIC: 4 µg ml^−1^; 2 *Aspergillus* spp. The ITR, VOR and POS MIC values were 2 and 8 µg ml^−1^, 8 and >32 µg ml^−1^ and 0.5 and 4 µg ml^−1^, respectively. Table 3 shows the isolates for which resistance was determined using BMD, agar plates and GT methods.

**Table 3.** AFST results determined by GT, agar plate and BMD for the isolates with MIC values resistant/highly resistant to azole group antifungals

*Specification*			GT			Agar plate			Microdilution			Mutations
***Sample No.***	**Sample type**	**Species**	**VOR**	**ITR**	**POS**	**VOR**	**ITR**	**POS**	**VOR**	**ITR**	**POS**	***Cyp51A* gene**
*H1*	**Tissue**	*A. fumigatus*	0.25	**1.5**	0.25	S	S	S	0.5	**2**	0.25	CypA-L98H and CypA-M220
*H2*	**Tissue**	*A. fumigatus*	**>32**	**4**	**0.75**	**R**	**R**	**R**	**>32**	**16**	**1**	CypA-L98H and CypA-M220
*H3*	**ETA**	*A. fumigatus*	0.125	**1.5**	0.094	S	S	S	0.25	**2**	0.064	CypA-L98H and CypA-M220
*H4*	**BAL**	*A. fumigatus*	0.19	1	0.064	S	S	S	0.25	1	0.032	CypA-L98H and CypA-M220
*H5*	**Sputum**	*A. terreus*	**1**	1	0.19	**R**	S	S	**0.5**	1	0.12	–
*H6*	**Tissue**	*A. niger*	0.38	**2**	0.012	S	**R**	S	0.5	**4**	0.12	CypA-L98H and CypA-M220
*H7*	**Corneal** **abscess**	*Aspergillus* spp*.*	**>32**	**4**	**0.5**	**R**	**R**	**R**	**>32**	**8**	0.5	CypA-M220
*H8*	**Tissue**	*Aspergillus* spp*.*	**1.5**	0.75	**8**	**R**	S	**R**	**8**	2	4	CypA-L98H and CypA-M220

### Species-level identification of *Aspergillus* spp. isolates resistance to azole antifungals by DNA sequence analysis

Clinical isolates of *A. fumigatus*, *A. niger* and *A. terreus* resistant to azole antifungal drugs identified by conventional methods and MALDI-TOF MS were also identified to species level by DNA sequence analysis (Fig. 1). Two isolates identified as *Aspergillus* spp. by conventional methods were identified as *Aspergillus pseudoglaucus*, a cryptic species, by sequence analysis.

### Mutations in the *Cyp51A* gene

In adapting the traditional PCR assay to SYBR Green-based real-time PCR with melting curve analysis, due to the diverse nature of the mutations, adaptation was evaluated with a two-step approach: size-based polymorphism (TR34/L98H) and single nucleotide polymorphisms (in M220).

Determination of TR34/L98H by melting curve: the melting curve is converted into a differential plot (−dF/dT – temperature). A single peak at a lower *T*_m_ (~80–82 °C) indicates a wild-type genotype. A single peak at a higher *T*_m_ (~84–86 °C) indicates a homozygous TR34/L98H genotype. Two distinct peaks indicate a heterozygous sample containing both alleles.

Detection of M220 point mutations by allele-specific qPCR: amplification is monitored by *Cq* (quantification cycle). A *Cq* value below a validated threshold value (e.g. *Cq*<35–38) in a given reaction indicates the presence of that allele. For example, early amplification in the M220T-specific tube, but not in the others, confirms the presence of the M220T mutation.

**Fig. 2.**
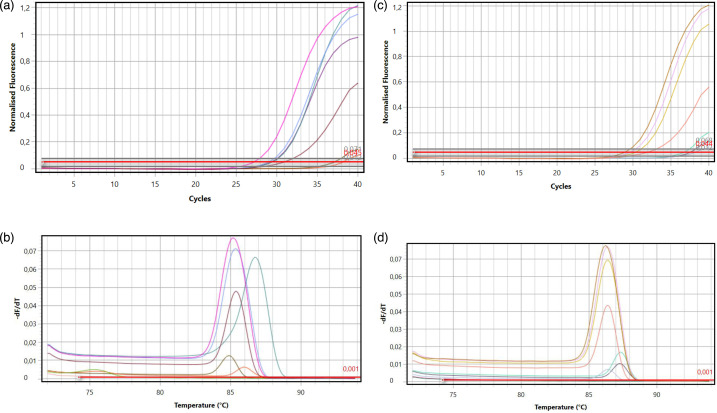
(a) L98-whole patient image bundle-syber green (cycling analysis), (b) L98-whole patient melt analysis image, (c) M220-whole patient image bundle-syber green (cycling analysis) and (d) M220-whole patient melt analysis images.

In this study, mutations in *cyp51A* were detected in clinical *Aspergillus* spp. isolates with resistance to azole antifungals. CypA-L98H point mutation in *cyp51A* was detected in six isolates, and CypA-M220 point mutation in seven isolates (Fig. 2). CypA-L98H and CypA-M220 mutations were not detected in the *A. terreus* isolate identified as *A. terreus*.

To evaluate the significance of these findings, Fisher’s exact test was applied to key comparisons. When comparing the efficacy of the agar plate method against the GT for detecting ITR resistance in *A. fumigatus*, the agar plate method correctly identified only one of the three GT-resistant isolates while correctly identifying all 52 susceptible isolates. This comparison yielded a *P* value of 0.0545, indicating that the observed 33.3% susceptibility of the agar plate method is of borderline statistical significance. Further statistical comparisons of resistance rates between species were limited by small sample sizes. The difference in ITR resistance between *A. fumigatus* (3 resistant, 52 susceptible) and *A. niger* (1 resistant, 3 susceptible) was not statistically significant (*P*=0.2506). Similarly, the difference in VOR resistance between *A. fumigatus* (1 resistant, 54 susceptible) and *A. terreus* (1 resistant, 5 susceptible) was not statistically significant (*P*=0.1885).

## Discussion

The major findings of this study of clinical *Aspergillus* isolates revealed an overall azole resistance rate of 5.6%, with *A. fumigatus* showing 1.8–5.45% resistance depending on the azole tested. Comparative evaluation of susceptibility testing methods showed that agar plate screening achieved 100% susceptibility and specificity for *non-fumigatus* species.

In our study, *A. fumigatus* (44%) was the most common species isolated from patients with aspergillosis. In the literature, Wang *et al*. reported *A. fumigatus* comprised 56.41% of *Aspergillus* isolates [15]. A US study of 2,138 isolates showed *A. fumigatus* was most prevalent at 96.91% [16].

The high percentage (56.8%) of isolates identified as *Aspergillus* spp. derived from respiratory tract samples was consistent with expectations, given that *Aspergillus* spp. are respiratory pathogens transmitted via airborne spores. In a study from Taiwan, a similar prevalence of *A. fumigatus* isolates was observed in respiratory tract specimens [17].

BMD and GT methods showed significant concordance for ITR-resistant *A. fumigatus* with 100% agreement for VOR and 93.3% for ITR, confirming GT as reliable for azole AFST [18]. Similar studies reported concordance rates of 99.1 and 87.8% for VOR and ITR [19], 97.6 and 95.8% [20] and 95 and 89% for VOR and POS [21].

*A. fumigatus* MIC50/MIC90 values increased significantly for azoles, with ITR showing the highest resistance rates [22]. BMD testing showed ITR MIC50/90 values of 0.5/1 µg ml^−1^ for *A. fumigatus*, *A. flavus* and *A. terreus* and 1/2 µg ml^−1^ for *A. niger* [23]. Another study reported higher ITR values for *A. fumigatus* and *A. niger* [24]*.* Jørgensen *et al*. found MIC50 ranges of 0.25–1 µg ml^−1^ for VOR, ≤0.125–1 µg ml^−1^ for ITR and 0.06–0.5 µg ml^−1^ for POS across *Aspergillus* species [25]. Our MIC50/90 values are consistent with those reported in the literature.

Mortality is significantly higher in patients with azole-resistant *A. fumigatus* compared to azole-sensitive infections [26,27]. Early detection of azole resistance is crucial. While reference BMD methods exist through CLSI and EUCAST guidelines, their complexity and time requirements create demand for simpler, faster, more cost-effective susceptibility testing methods [10,12].

The EUCAST standard agar plate method was designed for the phenotypic detection of ITR, VOR or POS resistance in * A. fumigatus* isolates in routine clinical mycology laboratories to obtain reliable results. Guinea *et al*. reported that the screening procedure enables rapid detection of azole resistance in clinical *A. fumigatus* isolates, facilitating earlier appropriate treatment [10]. Berkow *et al*. emphasized that the availability of a simple agar growth test for screening azole-resistant *Aspergillus* could make susceptibility testing more widespread in their review of current approaches to AFST [28].

Despite the detection of resistance to all azole antifungals by both the GT and agar plate method, the agar plate method failed to detect ITR resistance in the two *A. fumigatus* isolates. This is despite the fact that the MIC values were 1.5 µg ml^−1^ by the GT and 2 µg ml^−1^ by BMD. One *A. fumigatus* isolate exhibited a VOR MIC value of 0.25 µg ml^−1^ by GT and 0.5 µg ml^−1^ by BMD, while the POS MIC value was 0.25 µg ml^−1^ by both GT and BMD. The susceptibility and specificity for *A. fumigatus* were 100% for VOR and POS and 33.3 and 100% for ITR, respectively. The susceptibility of *A. flavus* to ITR was 100%, whereas that of *A. terreus* to both ITR and POS was 100%. Clinically significant L98H and M220 mutations in the *A. fumigatus cyp51A* gene, the azole target, are known to confer resistance [13]. The L98H mutation, often combined with a promoter tandem repeat (TR₃₄/L98H), causes broad pan-azole cross-resistance [29]. This mechanism is linked to environmental fungicide use, enabling primary resistant infections in azole-naïve patients [30]. Similarly, M220 point mutations (e.g. M220I/T/V) are key drivers associated with elevated MICs and cross-resistance to all azoles [31]. Furthermore, the presence of CypA-L98H and CypA-M220 mutations in the *Cyp51A* gene region of *Aspergillus* spp. isolates, which are most frequently associated with azole resistance, was detected in the present study. However, the agar plate method was ineffective in detecting resistance in * A. fumigatus* isolates carrying CypA-L98H and CypA-M220 mutations. When the agar plate method designed for *A. fumigatus* was applied to both *A. fumigatus* and *non-fumigatus Aspergillus* species, isolates with high MIC were successfully identified. However, this method tended to overlook *A. fumigatus* isolates with lower MIC values. The low susceptibility of *A. fumigatus* indicates that agar plate drug concentrations should be updated according to the latest revisions of the EUCAST guidelines. Resolution of the uncertainty in the use of agar plate screening tests for azole antifungals in *non-fumigatus Aspergillus* species will facilitate the detection of azole-resistant *Aspergillus* isolates. Arendrup *et al*. achieved 99% susceptibility/specificity using azole agar screening for resistant *A. fumigatus* with *Cyp51A* mutations (G54, N284, M220), missing only one M220T mutant that showed low MIC values [12].

According to EUCAST E.Def 10.1, of 322 *A. fumigatus* isolates, 30 (9.3%) were azole-resistant and 10 (3.1%) were non-wild-type. Three isolates showed ITR cross-resistance with POS and/or VOR resistance. All ten isolates were resistant to ITR, POS and VOR, with eight carrying *Cyp51A* mutations [25].

Serrano-Lobo *et al*. reported 100/93.3% susceptibility/specificity for agar screening in clinical *A. fumigatus* and cryptic species, concluding it effectively distinguishes azole-susceptible from azole-resistant *A. fumigatus* isolates. However, the method was less effective against cryptic species [32]. Accordingly, as stated in E. Def. 9.3.1, for isolates exhibiting reduced susceptibility to azoles, there is a requirement for corroboration using a reference BMD test [33]. Additionally, reference MIC testing should be conducted to confirm resistance using an agar plate [34].

GT and BMD methods for *Aspergillus* spp. were interpreted using EUCAST procedures (E.Def 7.4, 9.4, 11.0) with clinical breakpoints and ECOFFs [9]. Recent EUCAST revisions (E.Def 11.0; 2024) lowered resistance breakpoints for ITR and VOR in *A. fumigatus* from 2 to 1 µg ml^−1^ and ITR in *A. flavus* and *A. terreus* from 2 to 1 µg ml^−1^ [35]. Discrepancies between agar screening and BMD methods are likely to result from outdated agar plate drug concentrations that haven't been updated to match current EUCAST breakpoints [9]. We believe that to obtain accurate results, it is necessary to update the drug concentrations used in the agar plate screening method for VOR, ITR and POS to align them with the current breakpoints.

The identification of *non-fumigatus Aspergillus* isolates using conventional methods is limited, and DNA sequencing is needed. However, the frequency and mechanism of azole resistance in these species remain unknown. For example, detection of azole resistance in *A. terreus* isolates, which are naturally resistant to amphotericin B, is of utmost importance. In our study, an *A. terreus* isolate showed elevated MIC values by GT and BMD methods and VOR resistance by agar plate screening. Given *A. terreus'* natural amphotericin B resistance, AFST is vital for appropriate antifungal selection.

*A. niger* has lower virulence with few reported cases worldwide [36]. The unequal distribution of isolates among species is due to the clinical rarity of species such as *A. terreus* and *A. niger*. In our study, an *A. niger* isolate showed high ITR MIC values and carried CypA/L98H and CypA/M220 mutations in *Cyp51A*, with ITR resistance confirmed by the agar plate method.

Limited data on *non-fumigatus Aspergillus* species emphasize the necessity of AFST for these organisms. Since triazoles are the primary treatment for invasive infections, monitoring resistant isolate development and spread is essential. Periodic surveillance studies should track *Aspergillus* species distribution and resistance rates in clinical samples [5,6]. Conducting AFST on *non-fumigatus Aspergillus* isolates is important. Therefore, there is a clear need for additional data on these species. We believe that it is necessary to investigate the applicability of azole-containing agar plate screening tests for *non-fumigatus Aspergillus* isolates.

Our findings represent a significant contribution to regional surveillance in Turkey. This dataset helps fill a local data gap, as periodic surveillance studies are essential for tracking *Aspergillus* species distribution and resistance rates. These results have direct implications for antifungal stewardship in clinical laboratories. In contemporary practice, resistance in *Aspergillus* spp., particularly azole resistance emerging in cryptic species, can pose significant challenges during treatment. While our study showed the agar plate method was highly effective for the *non-fumigatus* isolates tested, its utility for the diverse range of cryptic species remains an area requiring further investigation. There are several limitations to this study. First, it was a single-centre study. Although the recommended gold standard for resistance testing is the BMD method, the GT was utilized as the primary method in this study. The high concordance rates between the GT and BMD in detecting azole resistance, along with the practical advantages of the GT, made this the preferred method for this study. A limitation of the study was the inability to include azole-resistant or susceptible *Aspergillus* spp. control strains for quality control.

## Conclusion

This study found 5.6% azole resistance prevalence in *Aspergillus* spp*.* with CypA-L98H and CypA-M220 mutations in *Cyp51A* detected. Azole resistance in clinical *Aspergillus* emphasizes the need for rapid susceptibility testing. Agar plate screening with updated breakpoints can facilitate early appropriate treatment. Reliable azole resistance detection in *non-fumigatus Aspergillus* isolates and establishing susceptibility/specificity for all *Aspergillus* species using agar screening may improve resistance monitoring and treatment approaches.

## Abbreviations

AFST: antifungal susceptibility testing
BAL: bronchoalveolar lavage
BMD: broth microdilution
ECOFFs: epidemiological cut-off values
ETA: endotracheal aspiration
GT: gradient test
ITR: itraconazole
PDA: potato dextrose agar
POS: posaconazole
qPCR: quantitative real-time PCR
SDA: Sabouraud dextrose agar
VOR: voriconazole
